# 

**Published:** 1877-05

**Authors:** 


### American Medical Association.

PHILADELPHIA, 1400 PINE STREET,
S. W. corner Bread.

The Twenty-eighth Annual Session will be held in the city of Chicago, Ill., on Tuesday, June 5th, 1877, in Farewell Hall, at 11 A. M.

"The delegates shall receive their appointment from permanently organized State Medical Societies, and such County and District Medical Societies as are recognized by *representation in their respective State Societies*, and from the Medical Department of the Army and Navy of the United States."

"Each State, County, and District Medical Society entitled to representation shall have the privilege of sending to the Association one delegate for every ten of its regular resident members, and one for every additional fraction of more than half that number: *Provided*, however, that the number of delegates for any particular State, territory, county, city, or town shall not exceed the ratio of one in ten of the resident physicians who may have signed the Code of Ethics of the Association."

Secretaries of Medical Socities as above designated are earnestly requested to forward *at once*, lists of their delegates. Will you kindly send to the undersigned a list of your members with their residences, in order that a correct record may be made of all who are in affiliation with this body?

"SECTIONS.—The chairmen of the several sections shall prepare and read in the general sessions of the Association, papers on the advances and discoveries of the past year in the branches of science included in their respective sections. * * * *"—BY-LAWS, Art. II., Sec. 4.

"Papers appropriate to the several sections, in order to secure consideration and action, must be sent to the Secretary of the appropriate section at least one month before the meeting which is to act upon them. It shall be the duty of the Secretary to whom such papers are sent, to examine them with care, and with the advice of the Chairman of his Section, to determine the time and order of their presentation, and give due notice of the same. * * * *"—BY-LAWS, Art. II., Sec. 5.

The following Committees are expected to report: On "Influence of Climate on Pulmonary Diseases in Florida,"—Dr. E. T. Sabal, Fla., Chairman. On "Animal Vaccination,"—Dr. Henry A. Martin, Mass., Chairman. On "The Inheritance of Syphilis,"—Dr. J. W. Thompson, Ky., Chairman. On "Prize Essays,"—Dr. N. S. Davis, Ill., Chairman. On "Necrology,"—Dr. S. C. Chew, M. D., Chairman. On "Catalogue of National Library,"—Dr. H. C. Wood, Pa., Chairman. WM. B. ATKINSON, M. D., Permanent Secretary.

### Books and Pamphlets Received.

Civil Malpractice: A Treatise on Surgical Jurisprudence, with chapters on Skill in Diagnosis, Treatment, Prognosis in Fractures. By Milo A. McClelland, M. D. New York: Hurd & Houghton. 8 vo., pp. 554. Price, $3 50.

Myelitis of the Anterior Horns, or Spinal Paralysis of the Adult and Child. By E. C. Seguin, M. D. New York: G. P. Putnam's Sons, 1877. Buffalo: Peter Paul.

Micro-Photographs in Histology, Normal and Pathological. By Carl Seiler, M. D., in conjunction with J. Gibbons Hunt, M. D., and Joseph G. Richardson, M. D. Philadelphia: I. H. Coates & Co. London; Macmillan & Co. Vol. I., No. 8.

American Journal of Insanity. Edited by Medical Officers of the New York State Lunatic Asylum, Utica. Vol. XXXIII., No. 4.

Extract from the Tenth Annual Report of the State Board of Charities of the State of New York, relating to the Cause of Pauperism. By Charles S. Hoyt, Sec. of Board. Transmitted to the Legislature, January 18, 1877.

Wine in the different forms of Anæmia and Atonic Gout. By M. E. Begin. Translated from the French. Paris: J. B. Bailliere & Sons, Rue Hautefeuïlle, 1877.

The Mortality of Surgical Operations in the Upper Lake States compared with that of other Regions. By Edmund Andrews, A. M., M. D., assisted by Thomas B. Lacey, M. D. Chicago, 1877.

Report of the Trustees of the Rhode Island Hospital, Presented to the Corporation at their Thirteenth Annual Meeting, Nov. 8, 1876.

The Popular Science Monthly. Conducted by E. L. Youmans. New York: D. Appleton & Co. May, 1877.

A Series of American Clinical Lectures. Edited by E. C. Seguin, M. D. Vol. III, No. 1. Transfusion of Blood and its Practical Application. By Thomas G. Morton, M. D.

### Contents of May Number.

### ORIGINAL.

THE BUFFALO

### Medical and Surgical Journal

PUBLISHED MONTHLY,

**Containing Original Articles, Reports of Medical Societies and Hospitals, Editorial, Reviews, Correspondence, Army News, etc., etc ; including the usual variety of Medical Periodical Publications. Specimen copies sent on application.**

Address Buffalo Medical & Surgical Journal, Buffalo, N. Y.

TERMS OF SUBSCRIPTION, $3.00 PER YEAR, IN ADVANCE.

